# Transcranial cortico-cortical paired associative stimulation (ccPAS) over ventral premotor-motor pathways enhances action performance and corticomotor excitability in young adults more than in elderly adults

**DOI:** 10.3389/fnagi.2023.1119508

**Published:** 2023-02-16

**Authors:** Sonia Turrini, Naomi Bevacqua, Antonio Cataneo, Emilio Chiappini, Francesca Fiori, Matteo Candidi, Alessio Avenanti

**Affiliations:** ^1^Centro Studi e Ricerche in Neuroscienze Cognitive, Dipartimento di Psicologia, Alma Mater Studiorum Università di Bologna, Cesena, Italy; ^2^Precision Neuroscience and Neuromodulation Program, Gordon Center for Medical Imaging, Massachusetts General Hospital and Harvard Medical School, Boston, MA, United States; ^3^Dipartimento di Psicologia, Sapienza Università di Roma, Rome, Italy; ^4^Department of Clinical and Health Psychology, University of Vienna, Vienna, Austria; ^5^Dipartimento di Medicina, NeXT: Unità di Ricerca di Neurofisiologia e Neuroingegneria dell’Interazione Uomo-Tecnologia, Rome, Italy; ^6^Centro de Investigación en Neuropsicología y Neurociencias Cognitivas, Universidad Católica del Maule, Talca, Chile

**Keywords:** TMS, ccPAS, Hebbian plasticity, manual dexterity, aging, motor system

## Abstract

Transcranial magnetic stimulation (TMS) methods such as cortico-cortical paired associative stimulation (ccPAS) can increase the strength of functional connectivity between ventral premotor cortex (PMv) and primary motor cortex (M1) *via* spike timing-dependent plasticity (STDP), leading to enhanced motor functions in young adults. However, whether this STDP-inducing protocol is effective in the aging brain remains unclear. In two groups of young and elderly healthy adults, we evaluated manual dexterity with the 9-hole peg task before and after ccPAS of the left PMv-M1 circuit. We observed that ccPAS enhanced dexterity in young adults, and this effect was anticipated by a progressive increase in motor-evoked potentials (MEPs) during ccPAS administration. No similar effects were observed in elderly individuals or in a control task. Across age groups, we observed that the magnitude of MEP changes predicted larger behavioral improvements. These findings demonstrate that left PMv-to-M1 ccPAS induces functionally specific improvements in young adults’ manual dexterity and an increase in corticomotor excitability, but altered plasticity prevents the effectiveness of ccPAS in the elderly.

## Introduction

Plasticity refers to the brain’s ability to change its structure and function in response to experience, a characteristic that persists well beyond infancy. Yet, during aging, progressive neuronal dysfunctions may lead to reduced plasticity (Burke and Barnes, 2006; Mahncke et al., 2006; Bhandari et al., 2016), potentially contributing to functional decline. For example, in the domain of motor control, older adults consistently show reduced manual dexterity and speed (Ranganathan et al., 2001; Carment et al., 2018). Although part of this impairment may result from peripheral changes, affecting, for instance, muscles or nerves, evidence also shows reduced white matter volume and density (Good et al., 2001; Resnick et al., 2003; Cox et al., 2016) and altered cortico-cortical interactions within premotor-motor networks in aging adults (Hinder et al., 2012; Green et al., 2018; Rurak et al., 2021; Verstraelen et al., 2021). Reduced manual performance in daily activities that involve object grasping and manipulation may reflect altered neural mechanisms within the dorsolateral visuomotor stream, particularly between the ventral premotor cortex (PMv) and the primary motor cortex (M1), which are key sensorimotor areas instrumental to transforming the intrinsic geometric properties of an observed object into appropriate motor commands (Davare et al., 2011; Rizzolatti et al., 2014; Fiori et al., 2018). Yet, whether younger and older adults show different sensitivities to exogenous inductions of plasticity in PMv-M1 connectivity is a relevant and entirely unexplored research question. To fill this gap, here, we used transcranial magnetic stimulation (TMS) to induce Hebbian associative plasticity in the PM-M1 network and investigate its effects on corticomotor excitability and manual motor performance in healthy elderly and young adults.

We used a TMS protocol called cortico-cortical paired associative stimulation (ccPAS), which is based on the Hebbian principle of associative plasticity. The ccPAS protocol involves repeatedly applying pairs of TMS pulses over two interconnected brain sites (Rizzo et al., 2009; Koch et al., 2013; Romei et al., 2016; Chiappini et al., 2018; Di Luzio et al., 2022) using an optimal interstimulus interval (ISI) between the pulses so that, for each TMS pair, the pulse administered over the first site (containing “pre-synaptic neurons” according to the Hebbian principle) would induce activity that spreads to the second site (containing “post-synaptic neurons”) immediately before or simultaneously with the TMS pulse over that second site. This pre- and post-synaptic coupling mimics patterns of neural stimulation instrumental to achieving spike timing-dependent plasticity (STDP) (Caporale and Dan, 2008; Markram et al., 2011), thus enhancing (or weakening) the strength of the neural pathway connecting the stimulated brain areas.

Studies have shown that ccPAS can be used to induce STDP in the PMv-to-M1 pathway, leading to enhanced corticomotor excitability and network efficiency (Buch et al., 2011; Johnen et al., 2015; Fiori et al., 2018; Chiappini et al., 2020; Sel et al., 2021; Casarotto et al., 2022; Turrini et al., 2022); in particular, studies have shown that PMv-M1 ccPAS can enhance hand function and corticomotor excitability in young adults (Buch et al., 2011; Fiori et al., 2018; Casarotto et al., 2022). Moreover, consistent with the Hebbian principle, prior studies have shown that no similar enhancement is observed when reversing the order of the pulses or administering sham ccPAS (Buch et al., 2011; Fiori et al., 2018; Turrini et al., 2022). However, none of the previous studies have tested whether Hebbian plasticity can be induced in elderly adults using ccPAS. This is a potentially relevant question to scrutinize, as testing ccPAS efficacy in the aging brain would stimulate clinical investigation of this protocol in aging-related pathological conditions such as neurodegenerative disorders.

To test whether enhanced efficiency of the PMv-to-M1 pathway could be obtained in older individuals and explore the relationship between physiological indices of STDP and manual dexterity, here, we administered ccPAS over the left PMv-to-M1 circuit in a sample of healthy young and elderly adult participants and assessed changes in manual dexterity after stimulation.

## Materials and methods

### Participants

We tested 28 healthy volunteers, divided into two groups of 14 individuals each based on their chronological age (Table 1). This sample size was based on a power calculation computed in Gpower, using a power (1-β) of 0.80 and an alpha level of 0.05, two-tailed. Assuming a medium/large effect size (*f* = 0.32), based on previous results that used a similar ccPAS protocol in healthy young adults (Fiori et al., 2018), the suggested sample size was 24 participants. We increased the sample size to 28 to account for possible attrition or technical failures. All participants were right-handed based on the Edinburgh Handedness Inventory (Oldfield, 1971) (mean score 88.5 ± 20.8), had normal or corrected-to-normal vision and were naïve to the purpose of the experiment. All participants gave written informed consent prior to the study, and were screened to avoid adverse reactions to TMS (Rossi et al., 2021). Older participants were not cognitively impaired, as indexed by the Mini Mental State Examination (MMSE, mean corrected score 27.1 ± 0.2, range 24.2–28.4) and the Raven’s colored progressive matrices (mean corrected score 29.6 ± 0.5, range 29–39), and they had adequate power grip and precision grip strengths, as assessed by a force transducer. None of the participants reported adverse reactions or discomfort related to TMS. Physiological data (motor-evoked potentials, MEPs) from one elderly participant were excluded due to technical failure. All analyses were conducted on 14 young adults and 13 older adults, including analyses of behavioral data. Importantly, all statistical results observed in the behavioral data were fully replicated when including the older participant with no physiological data.

**TABLE 1:** Demographic information, neurophysiological parameters and motor performance of the two groups at Baseline.

	Elderly	Young	Stat. analyses
Age: Mean year ± SD	72 ± 6 y	24 ± 3 y	*t*_25_ = 24.98, *p* = 0.02
rMT: Mean max stimulator output ± SD	57 ± 17%	43 ± 9%	*t*_25_ = 2.71, *p* = 0.01
1 mV intensity: Mean max stimulator output ± SD	70 ± 18%	66 ± 15%	*t*_25_ = 0.61, *p* = 0.54
Baseline 9HPT: Mean execution time in s ± SD	31 ± 7 s	22 ± 2 s	*t*_25_ = 5.09, *p* < 0.001
Baseline cRT: Mean execution time in ms ± SD	597 ± 139 ms	392 ± 24 ms	*t*_25_ = 5.45, *p* < 0.001
Baseline cRT accuracy: Mean% of correct resonses ± SD	96 ± 7%	96 ± 3%	*t*_25_ = −0.05, *p* = 0.96

### Procedure

**FIGURE 1:**
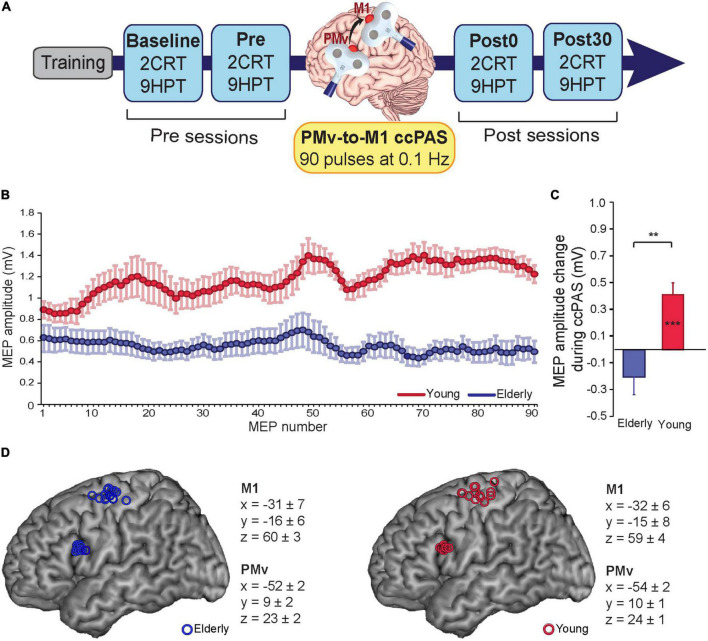
**(A)** Experimental design. **(B)** motor-evoked potentials (MEPs) during cortico-cortical paired associative stimulation (ccPAS) in elderly adults (blue) and young adults (red). **(C)** MEP modulation index in the two groups (the last 10 MEPs relative to the first 10 MEPs acquired during ccPAS). **(D)** Individual participants’ targeted sites reconstructed onto a standard template using icbm2tal after conversion to MNI space. Error bars represent standard error of the mean; **p ≤ 0.01; ***p ≤ 0.001.

To evaluate changes in manual dexterity after inducing plasticity in the PMv-M1 pathway, participants performed an experimental task, i.e., the 9-Hole Peg Test (9HPT), and a choice reaction task (cRT) as a visuomotor control task (for tasks details, see the next paragraph). After a brief training phase (∼10 min), participants were asked to perform the two tasks at four timepoints (Figure 1A): two before ccPAS (“Baseline” and “Pre” sessions), one immediately after (“Post0”) and one 30 min after ccPAS (“Post30”). Each session lasted ∼5 min, during which the two tasks were administered in a counterbalanced order across participants. Sessions were separated by a rest period of ∼25 min. The experimental procedure (lasting approximately 2.5 h) was in accordance with the Declaration of Helsinki and approved by the Bioethics Committee of the University of Bologna.

### Behavioral tasks

The 9HPT is widely used to assess fine dexterity in the hand. It requires participants to finely shape their hand in order to grasp and manipulate small objects (Mathiowetz et al., 1985; Oxford Grice et al., 2003); thus, it is thought to rely on activation of the dorsolateral stream (Grol et al., 2007; Davare et al., 2010). Indeed, performance on the 9HPT correlates with recruitment of sensorimotor areas, including PMv and M1 (Hamzei et al., 2012). Critically, this task was found to be sensitive to non-invasive manipulations of the motor system (Koch et al., 2008; Avenanti et al., 2012b), including the strength of the PMv-to-M1 pathway (Fiori et al., 2018). The 9HPT apparatus consisted of a plastic board with 9 small holes organized in a 3 × 3 matrix. Upon receiving the start command, participants pressed the space bar on a nearby laptop to start a clock, picked up the nine small pegs, put each peg into one of the nine holes with their right hand, one at the time, then removed them one by one, returned them to the box, and pressed the same space bar to stop a clock and record their performance time. Participants were required to execute the task as quickly as possible. Participants performed 5 repetitions of the task at each timepoint (Baseline, Pre, Post0, Post30).

The cRT was used as a visuomotor control task. We used a 2-choice version of the cRT to assess simple visuomotor mapping based on learned associations. We selected this task because, similarly to the 9HPT, the cRT requires visuomotor transformation and shows sensitivity to TMS of M1 (Kobayashi et al., 2004; Mansur et al., 2005). Crucially, however, the cRT task does not involve object grasping and manipulation, which relies on PMv integrity (Davare et al., 2006, 2011) and PMv-to-M1 connections (Fiori et al., 2018). Thus, we expected that cRT performance would not be affected by modulation of PMv-to-M1 pathway connectivity, in line with prior observations (Fiori et al., 2018). Participants were instructed to respond by releasing a key pressed by the index or middle finger of the right hand according to which number (‘1’ or ‘2’) was displayed with equal probability on a monitor placed ∼80 cm in front of them. Participants were instructed to perform the task as quickly and accurately as possible. Each task consisted of 40 trials. Task accuracy (% of correct response) and mean reaction times (RTs) of correct responses were collected for each session.

### ccPAS protocol

The ccPAS pulses were administered by means of two figure-of-eight branding iron coils (inner coil diameter of 50 mm) connected to two Magstim 200^2^ monophasic stimulators (Magstim Company, Carmarthenshire, Wales, UK). These small focal coils are designed with the handle pointing perpendicular to the plane of the wings and could be positioned near to each other without interference from the handles. Ninety pairs of TMS pulses were delivered continuously at a rate of 0.1 Hz for 15 min (Rizzo et al., 2009; Buch et al., 2011; Johnen et al., 2015; Romei et al., 2016; Chiappini et al., 2018, 2020; Fiori et al., 2018); in each pair, PMv stimulation preceded M1 stimulation by 8 ms (Buch et al., 2011; Johnen et al., 2015; Fiori et al., 2018) to activate short-latency connections from PMv to M1 (Davare et al., 2008, 2009). The 0.1-Hz frequency was selected to be consistent with prior ccPAS studies conducted by both our group (Fiori et al., 2018, Turrini et al., 2022) and other research groups (Buch et al., 2011; Johnen et al., 2015; Sel et al., 2021); additionally, it allowed us to exclude the possibility that any observed effect produced by ccPAS might have been due to repeated stimulation of a single area, rather than manipulation of the synaptic efficacy of PMv-to-M1 connections, as 0.1 Hz stimulation was found to be ineffective at modulating the excitability of the stimulated cortical site (Chen et al., 1997).

PMv pulse intensity was set to 90% of the individual’s resting motor threshold (Fiori et al., 2018; Turrini et al., 2022), defined as the minimum stimulator output intensity able to induce MEPs > 50 μV in 5 out of 10 consecutive trials (Rossini et al., 2015). In all participants, the resting motor threshold (rMT) was assessed immediately before the ccPAS protocol. M1 pulse intensity was adjusted to evoke∼1 mV MEPs (Buch et al., 2011; Johnen et al., 2015; Fiori et al., 2018). This suprathreshold intensity allowed us to record MEPs during paired stimulation and measure corticomotor excitability changes online (Fiori et al., 2018; Turrini et al., 2022; Figure 1B). The pulses were triggered remotely using MATLAB (MathWorks, Natick, MA, USA) to control both stimulators. To minimize discomfort, before starting ccPAS, we exposed participants to active stimulation of the PMv using 3–4 pulses of increasing intensity. All participants reported that the stimulation was tolerable.

The coil positions to target the left PMv and left M1 were identified using established methods. While the hand representation in the left M1 was identified functionally based on MEPs from the right first dorsal interosseus (FDI) muscle (Rossini et al., 2015), the left PMv was identified using the SofTaxic Navigator System (Electro Medical System, Bologna, Italy) as in previous studies (Avenanti et al., 2007; Tidoni et al., 2013; Fiori et al., 2016, 2017, 2018; Paracampo et al., 2018). Skull landmarks (nasion, inion, and 2 preauricular points) and ∼80 points providing a uniform representation of the scalp were digitized by means of a Polaris Vicra digitizer (Northern Digital). An individual estimated magnetic resonance image (MRI) was obtained for each subject through a 3D warping procedure fitting a high-resolution MRI template to the participant’s scalp model and craniometric points. This procedure has been proven to ensure a global localization accuracy of roughly 5 mm (Carducci and Brusco, 2012). To target the left PMv, the coil was placed on a scalp region overlying the Talairach coordinates *x* = −52; *y* = 10; *z* = 24 (Fiori et al., 2018; Turrini et al., 2022). These coordinates were obtained by averaging previously reported coordinates (Davare, 2006; Dafotakis et al., 2008; Avenanti et al., 2012a,^2018^; Jacquet and Avenanti, 2015); those studies showed that stimulating this ventral frontal site (at the border between the anterior sector of the PMv and the posterior sector of the inferior frontal gyrus) affected planning, execution and perception of hand actions. These coordinates were also consistent with those used in TMS studies targeting PMv-to-M1 connections (Davare et al., 2008, 2009, 2010; Fiori et al., 2016, 2017; Zanon et al., 2018). The Talairach coordinates corresponding to the projections of the left PMv and left M1 scalp sites onto the brain surface were automatically estimated by the SofTaxic Navigator from the MRI-constructed stereotaxic template; the resulting Talairach coordinates in the two age groups can be found in Figure 1D. Coils were held to induce current flows consistent with previous dual-site TMS and ccPAS studies targeting PMv and M1 (Davare et al., 2008; Bäumer et al., 2009; Buch et al., 2011). The left PMv coil was placed tangentially to the scalp, inducing a posterior-to-anterior and lateral-to-medial current flow in the brain pointing toward the M1 coil, in keeping with prior dual coil and ccPAS studies targeting the PMv-M1 circuit (e.g., Davare et al., 2008; Buch et al., 2011; Fiori et al., 2018). The left M1 coil was placed was placed tangentially to the scalp and oriented at a ∼45 angle to the midline, inducing a posterior-to-anterior current flow optimal for M1 stimulation (Kammer et al., 2001). This dual coil configuration is proposed to recruit presynaptic inputs from PMv to pyramidal cells located in layer 5 of M1 (Casarotto et al., 2022).

During the ccPAS protocol, participants remained relaxed with their eyes open, and MEPs were recorded from the right FDI by means of surface Ag/AgCl electrodes placed in a belly-tendon montage, with the ground electrode placed on the right wrist. EMG signals were acquired by means of a Biopac MP-35 electromyograph (Biopac, USA), band-pass filtered (30–500 Hz) and digitized at a sampling rate of 5 kHz. EMG traces were stored for the analysis of MEPs recorded online during ccPAS. Peak-to-peak amplitudes of each MEP were assessed. MEPs too small (≤50 μV) or preceded by EMG activity deviating ≥2SD from the participant’s rectified mean were discarded. The remaining MEPs (89% of total trails) were smoothed through a sliding average with a 7-trial window width (Figure 1B).

### Data analyses

Mean 9HTP and cRT performance indices (i.e., 9HPT execution time, cRT accuracy, and cRT speed) were computed for each session and compared at Baseline between groups using an analysis of variance (ANOVA). To account for Baseline differences between groups and normalize the data distributions, 9HTP, and cRT performance indices in the Pre, Post0, and Post30 sessions were expressed as% of Baseline and then submitted to Age (young, elderly) x Time (Pre, Post0, Post30) ANOVAs, one for each behavioral metric. *Post hoc* analyses were conducted using Duncan’s tests. MEPs were assessed by measuring peak-to-peak EMG amplitude (in mV). A MEP modulation index was computed as the difference between the last and the first 10 MEPs, and compared between groups using an ANOVA (Figure 1C). To investigate whether neurophysiological indices of Hebbian plasticity predicted the magnitudes of behavioral changes following ccPAS in the two groups, we used general regression models with MEP modulation during ccPAS and its interaction with age as predictors of ccPAS-induced behavioral changes in the 9HPT at (i) Post0 and (ii) Post30 timepoints.

## Results

Table 1 shows that, at Baseline (i.e., before ccPAS), younger participants showed better motor performance than elderly participants, with faster execution times in the 9HPT (*p* < 0.001, *Cohen’s d* = 1.93) and in cRT RTs (*p* < 0.001, *Cohen’s d* = 2.06), but comparable cRT accuracy (*p* > 0.96). Elderly participants had higher rMTs than younger participants (*p* < 0.001), whereas the two groups did not differ in the intensity necessary to induce MEPs with an amplitude of about 1 mV (*p* = 0.54).

During ccPAS, young participants showed a gradual enhancement of MEPs that accurately fit a linear distribution [*f*(*x*) = 0.0048*x + 0.964; *R^2^_*adj*_* = 0.68], whereas no consistent change was observed in older individuals (see Figure 1B). Figure 1C shows that young participants had larger MEPs at the end of ccPAS than at the beginning (*F*_1,13_ = 21.48, *p* = 0.0005, η_*p*_^2^ = 0.62), whereas no difference between MEPs the end and the beginning of the protocol was observed in elderly participants (*F*_1,12_ = 2.46, *p* = 0.14, η_*p*_^2^ = 0.16); moreover, changes in MEPs were larger in young participants than in elderly participants (*F*_1,25_ = 7.06, *p* = 0.013, η_*p*_^2^ = 0.22).

**FIGURE 2:**
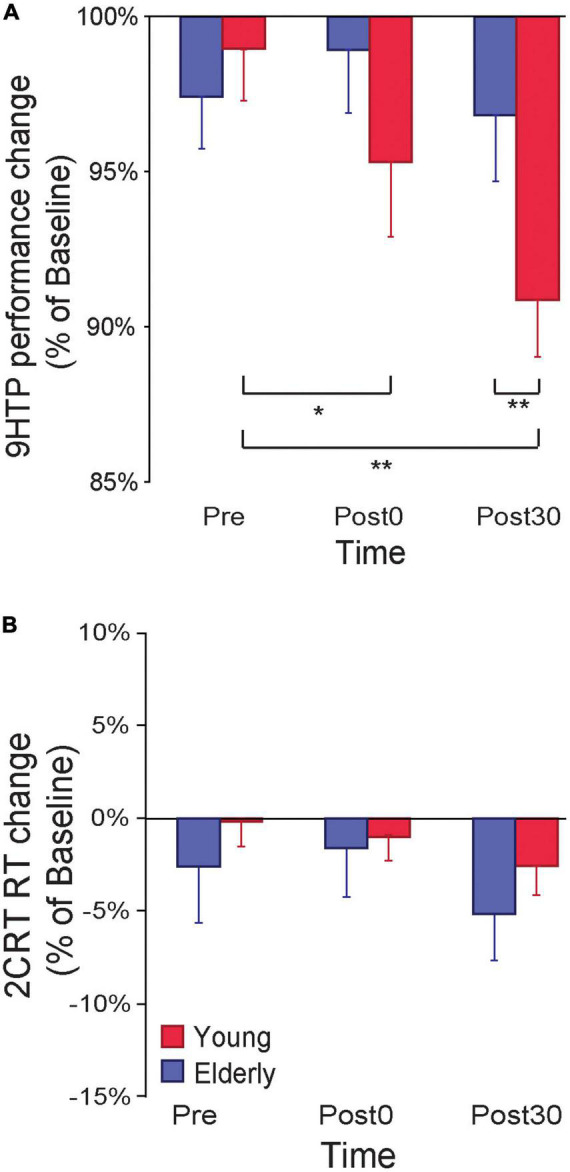
**(A)** 9-Hole Peg Test (9HPT) performance improved following PMv-to-M1 cortico-cortical paired associative stimulation (ccPAS) in young but not elderly participants. **(B)** The ccPAS manipulation did not affect choice reaction task (cRT) performance in either group. Error bars represent standard error of the mean; *p ≤ 0.05, **p ≤ 0.01.

An ANOVA on 9HPT performance ratios (% of Baseline) with the between-subjects factor Age (young, elderly) and the within-subjects factor Time (Pre, Post0, Post30) showed a main effect of Time (*F*_2,50_ = 11.53, *p* < 0.001, η_*p*_^2^ = 0.31), qualified by a significant Age*Time interaction (*F*_2,50_ = 8.12, *p* < 0.001, η_*p*_^2^ = 0.24; Figure 2A). Young participants showed a reduction in 9HPT execution time following ccPAS (Post0: 95% ± 7%, *p* = 0.015, *Cohen’s d* = 0.68; Post30: 91 ± 6%, *p* = 0.002, *Cohen’s d* = 1.54), relative to pre-ccPAS levels (Pre: 98 ± 6%). In contrast, we found no performance improvement in older participants (Pre: 97 ± 6%; Post0: 98 ± 7%; Post30: 97 ± 7%; all *p* ≥ 0.25). Furthermore, while performance did not differ between groups at Pre (*p* = 0.66), and Post0 (*p* = 0.20), it was significantly different at Post30 (*p* = 0.037, *Cohen’s d* = 0.88), indicating that PMv-to-M1 ccPAS improved hand dexterity in young participants only, with larger effects 30 min after the end of the ccPAS protocol.

A similar Age x Time ANOVA on cRT performance (% of Baseline) showed no main or interaction effects on accuracy (all *F* ≤ 0.51, *p* ≥ 0.61) or speed (all *F* ≤ 2.70, *p* ≥ 0.08; see Figure 2B).

**FIGURE 3:**
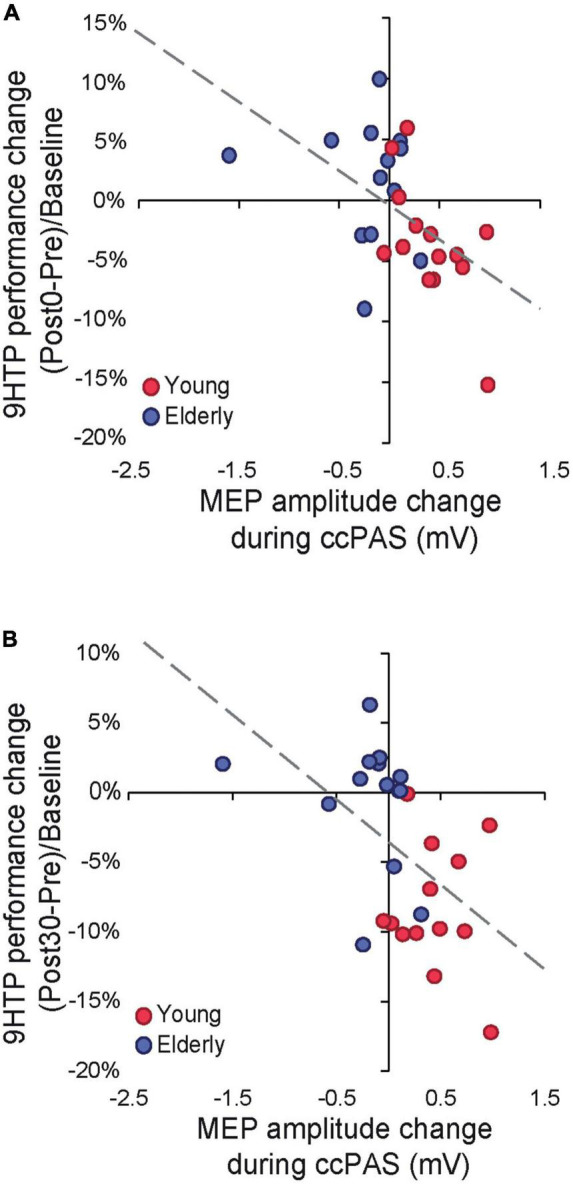
**(A)** Cortical plasticity predicts 9-Hole Peg Test (9HPT) performance changes following cortico-cortical paired associative stimulation (ccPAS) at Post0. **(B)** Cortical plasticity predicts 9HPT performance changes following ccPAS at Post30.

Finally, we tested whether neurophysiological indices of Hebbian plasticity predicted changes in behavior following ccPAS. We carried out two regression models testing the MEP modulation index and its interaction with age as predictors of 9HPT performance changes at Post0 and Post30. Both models were significant (Post0: *R*^2^*_*adj*_* = 0.31; Post30: *R*^2^*_*adj*_* = 0.23; all *F* ≥ 4.89, *p* ≤ 0.017, η_*p*_^2^ ≥ 0.29), showing that only MEP modulation predicted the magnitude of 9HPT speed increases at Post0 (β = −0.54, *p* = 0.003; Figure 3A) and Post30 (β = −0.53, *p* = 0.005; Figure 3B).

## Discussion

Repeatedly administering TMS to PMv prior to M1 evokes synchronous pre- and postsynaptic activity in the PMv-to-M1 pathway, thus strengthening that network *via* STDP (Buch et al., 2011; Johnen et al., 2015; Fiori et al., 2018; Chiappini et al., 2020; Sel et al., 2021; Casarotto et al., 2022; Turrini et al., 2022). Our results indicate that, by strengthening PMv-M1 cortico-cortical connectivity, the ccPAS protocol effectively enhances 9HTP performance in young adults (Fiori et al., 2018), confirming the crucial role of PMv-M1 interactions in visually guided fine manual dexterity (Davare et al., 2011; Rizzolatti et al., 2014; Fiori et al., 2018). The behavioral enhancement was specific to an experimental task that taps into PMv-M1 functioning (i.e., the 9HPT) (Davare et al., 2011; Rizzolatti et al., 2014; Fiori et al., 2018), and was not observed in a control task that engages the PMv-M1 network to a lesser extent.

Remarkably, behavioral improvements were predicted by a progressive growth in MEP amplitude during ccPAS, such that individuals who displayed a greater increase in corticomotor excitability at the end of ccPAS (Figure 1C)–reflecting the malleability and enhanced efficiency of the targeted circuit (Turrini et al., 2022)–also showed stronger improvements in 9HPT performance (Figures 3A, B). The progressive nature of the plastic effects–already apparent in the neurophysiological modulation of MEP size during ccPAS, and building up at the behavioral level after the end of ccPAS–is consistent with the time course of Hebbian plasticity (Bi and Poo, 2001; Caporale and Dan, 2008) and LTP-like effects previously described in both the human motor system (Stefan et al., 2000; Ziemann et al., 2008) and the visual system (Romei et al., 2016; Chiappini et al., 2018; Chiappini et al., 2022, Di Luzio et al., 2022). Interestingly, behavioral enhancements increased in magnitude over time, with a smaller (although already fully significant) effect detected at Post0 and becoming more prominent at the Post30 timepoint, in keeping with other ccPAS studies showing similar temporal dynamics (Romei et al., 2016; Fiori et al., 2018; Di Luzio et al., 2022).

Neither behavioral nor neurophysiological changes were observed in older adults, in line with previous evidence of reduced synaptic plasticity in the aging brain (Burke and Barnes, 2006; Mahncke et al., 2006; Bhandari et al., 2016). Additionally, we replicated robust previous findings of reduced manual dexterity and speed in the elderly (Ranganathan et al., 2001; Burke and Barnes, 2006; Mahncke et al., 2006; Bhandari et al., 2016; Carment et al., 2018), and preserved accuracy (Forstmann et al., 2011). Although our elderly sample did not show a consistent improvement in dexterity on the 9HPT following ccPAS, the relation between increased motor excitability during the protocol and hand dexterity improvements was similar in both young and old participants–suggesting that preserved physiological indices of STDP predict behavioral improvements after ccPAS not only in young adults, but in the elderly as well. This further supports the link between plasticity and motor function. Thus, our findings expand prior work showing altered cortico-cortical connectivity in aging (Resnick et al., 2003; Hinder et al., 2012; Cox et al., 2016; Green et al., 2018; Rurak et al., 2021; Verstraelen et al., 2021) by highlighting a reduction in Hebbian plasticity within the PMv-M1 network.

Our study emphasizes potential challenges in applying protocols such as ccPAS to induce STDP in the aging brain. First, we found that older adults displayed reduced manual dexterity at Baseline and reduced plastic potential and responsiveness to ccPAS, compared with young adults; the relation between these two findings is unclear, and worthy of further inspection, to clarify whether reduced plasticity could be a contributing factor to functional decline in the elderly. If that was the case, an effort to find innovative and non-invasive methods to promote and facilitate plasticity in the aging brain would be of paramount relevance. To this aim, our findings raise the interesting question of how to adapt and personalize available non-invasive brain stimulation tools to the aging population. Indeed, in the present work, we employed a well-established ccPAS protocol (Buch et al., 2011; Johnen et al., 2015; Fiori et al., 2018; Turrini et al., 2022) which is informed by the timing and patterns of connectivity explored in healthy young adults (Davare et al., 2008, 2009), to repeatedly activate the targeted pathway in a way that is consistent with its physiological wiring. However, previous results indicate that connectivity in the motor systems of elderly adults may be characterized by disrupted cortico-cortical interactions (Green et al., 2018; Rurak et al., 2021); hence, the implementation of protocols adapted to this physiological shift would be advisable.

Therefore, our study calls for further research exploring the residual plastic potential of the aging brain and elucidating how to implement non-invasive brain stimulation to effectively promote plasticity in the healthy elderly population.

## Data availability statement

The raw data supporting the conclusions of this article will be made available by the authors, without undue reservation.

## Ethics statement

The studies involving human participants were reviewed and approved by Bioethics Committee of the University of Bologna. The patients/participants provided their written informed consent to participate in this study.

## Author contributions

ST: methodology, software, investigation, data curation, formal analysis, visualization, and writing—original draft. NB: investigation, data curation, and writing—review and editing. AC: investigation and data curation. FF: methodology, software, investigation, data curation, and writing—review and editing. EC: methodology, software, investigation, data curation, and writing—review and editing. MC: supervision, writing—review and editing, and funding acquisition. AA: conceptualization, formal analysis, supervision, project administration, funding acquisition, and writing—original draft. All authors contributed to the article and approved the submitted version.
